# “A laugh a day keeps the failure away”: The role of self-enhancing humor and host country community embeddedness in career satisfaction of dual-earner expatriate couples

**DOI:** 10.3389/fpsyg.2023.1125136

**Published:** 2023-03-01

**Authors:** Anh Nguyen, Maike Andresen

**Affiliations:** Department of Social Sciences, Economics and Business Administration, University of Bamberg, Bamberg, Germany

**Keywords:** career satisfaction, expatriate couples, humor, job embeddedness, actor-partner interdependence mediation model (APIMeM)

## Abstract

For dual-earner expatriate couples (DEECs), it is particularly challenging to achieve career satisfaction after relocating to another country and the associated career transitions. While studies have addressed the strenuous career pathways of individuals in DEECs, the identification and empirical analysis of resources that may contribute to the attainment of career satisfaction remains a desideratum. This research investigates the impact of self-enhancing humor and community embeddedness on individual career satisfaction and the crossover effects of humor on that of the partners in DEECs. Using data from 109 DEECs in Europe and Actor-Partner Interdependent Model (APIM), the results show that embeddedness in one’s community mediated the relationship between humor and career satisfaction. The study also unfolded the crossover effects of humor within couples: Men’s humor promoted their female partner’s community embeddedness, which, in turn, promoted women’s career satisfaction. This study entails fruitful implications for future research on DEECs and practical recommendations for relevant stakeholders to facilitate the careers of DEECs.

## Introduction

Relocating abroad is a strenuous career transition that makes the career satisfaction of couples particularly challenging in comparison to domestic dual-earner couples. Dual-earner expatriate couples (DEECs) refer to couples who relocate across national borders and who both earn an income in the host country. Career obstacles of DEECs include unrecognized qualifications, lack of networks, work permit restrictions, language deficiencies, and unfavorable attitudes of host country employers toward foreign workers (Mäkelä et al., 2011; Kierner, 2018). Previous studies demonstrate that at least one of the partners often suffers career interruptions after relocation (Taylor, 2007; Rabe, 2011; Krieger, 2020; Samper and Kreyenfeld, 2021), a decline in occupational status (Ballarino and Panichella, 2018; Kierner, 2018), or even career relinquishment (McNulty and Moeller, 2018; Kanstrén, 2021). DEEC partners experience significant stress at work caused by the new work conditions in their host country, including long hours, frequent business travel, and unfit jobs (Fischlmayr and Kollinger, 2010; Mäkelä et al., 2011; Kierner, 2018). However, in addition to confronting direct work-related career obstacles, DEECs are also known to face personal travails that exacerbate the challenges of individual career management. As a case in point, expatriate couples have to deal with additional domestic demands due to the relocation context, such as childcare, housework, and family settlement (Mäkelä et al., 2011; Khokher and Beauregard, 2014). Thus, in order to fulfill their duties and ambitions at work and at home, cohabiting DEEC partners must closely coordinate their career strategies and decisions (Rabe, 2011; Känsälä et al., 2015), leading to each partner playing an influential role in the other’s career satisfaction.

The current literature on DEECs’ career satisfaction is fraught with three shortcomings. First, the approach of viewing DEECs’ career satisfaction as a dyadic outcome is rarely found in existing research. In contrast, most studies on the dual-career satisfaction of DEECs focus on how to improve the career satisfaction of one partner, typically those sent to work abroad by their employers (i.e., assigned expatriates/AEs) or their trailing spouse (cf. Harvey, 1998; Selmer and Leung, 2003; Mäkelä et al., 2011; McNulty and Moeller, 2018). On one hand, this approach has overlooked each partner’s dyadic influences on the other’s career satisfaction (Mancini-Vonlanthen, 2021; Mayrhofer et al., 2021). On the other hand, there is paucity of evidence on different types of expatriate couples with different resource endowments, including those who relocate on their own initiative without an organizational relocation package (i.e., self-initiated DEECs) (Mayrhofer et al., 2021). Secondly, existing research mainly has a negative perspective and concentrates on the negative impacts like depletion of resources and stress that hinder DEECs’ career satisfaction. There is less information available on the resources that help DEECs in their career accomplishments (Kupka and Cathro, 2007; McNulty and Moeller, 2018). Consequently, there is insufficient evidence to build suitable practices and strategies for their career management. Thirdly, previous research on career satisfaction has particularly focused on proximal antecedents in the organizational context (e.g., Ho et al., 2022), leaving out the contextual conditions.

We aim to address these shortcomings by adopting a *positive approach* and by addressing *extra-organizational factors* that influence DEECs’ *dyadic* career satisfaction. Specifically, we explore two types of resources to determine their potential contribution to DEECs’ career satisfaction upon relocation: self-enhancing humor (SEH) and community embeddedness. SEH is a personality trait where an individual has the proclivity to find humor in the absurdities of life and use it as a way to handle stress and other predicaments (Martin et al., 2003). Community embeddedness captures the connectedness with the locality in the host nation (Mitchell et al., 2001). We propose that SEH promotes career satisfaction in DEECs by providing multifaceted resources for individuals to invest in achieving career goals and mitigating resource losses (Cooper et al., 2018) during career transitions. This proposal is premised on the Conservation of Resources (COR) theory, which posits that individuals must invest resources to achieve important goals (Hobfoll, 2001). SEH further facilitates satisfactory career pathways through community embeddedness, which entails essential resources (e.g., networks, cultural awareness, safety) to build a career abroad (Kanstrén and Suutari, 2021). We ask the following questions to examine the role of SEH in the career satisfaction of partners in DEECs: *To what extent does SEH enhance the career satisfaction of each partner in DEECs? How does community embeddedness impact the relationship between SEH and career satisfaction as a mediator?*

According to the COR theory, resources do not exist discretely but usually exist in packs or caravans, crossing over from one individual to another, particularly in the availability of suitable passageways (Hobfoll et al., 2018). The wheel model of humor postulates that partners with SEH potentially create a home environment that supports humor where they initiate and foster pleasant emotions, playful expressions and interactions, and eventually, a frequent playful and exhilarating climate (Robert and Wilbanks, 2012). This, in turn, provides an ideal passageway for the vital resources that humor produces–such as boosting emotions and work engagement–to be transferred within DEECs and promote career satisfaction for *both* partners (cf. Wijewardena et al., 2017; Zhang and Su, 2020). Gender seems to play a role in this process, as women are more likely than men to “catch” facial expressions and synchronize emotions with others. This leads to the following questions: *How does each partner’s SEH affect the other’s career satisfaction and community embeddedness as a mediator? How do these effects differ between women and men?*

The contributions of this study are threefold. First, even though the literature has demonstrated that pleasant emotions (e.g., happiness) and positive traits (e.g., proactivity) predict career satisfaction in the domestic population (Ng et al., 2005; Walsh et al., 2018), scholars barely adopt a positive approach to studying the careers of DEECs. Instead, the majority of studies on DEECs focus on negative emotions (e.g., frustration, anger, and hopelessness) and social isolation (Brown, 2008; Kierner, 2018; McNulty and Moeller, 2018). By adopting a positive approach, this study shifts the focus from strains to resources (i.e., from problem-focused to solution-focused) and identifies ways of promoting DEECs’ career satisfaction. Second, we quantify the influences of each partner’s resources on the other’s accomplishment, by using a dyadic research and analytical model. This article thus opens doors to view career satisfaction as a dyadic outcome among expatriate couples, encouraging future research in the same direction. The last contribution includes practical recommendations for organizations and expatriate couples who want to manage their dual careers more effectively.

The structure of this manuscript is as follows. The next section provides an overview of the relationships between SEH, community embeddedness, and career satisfaction, as well as the crossover effects between partners that form the basis of our hypotheses. We then elucidate the methodology, including the Actor-Partner Interdependence Model (APIM), and present the research results. This is followed by a discussion of theoretical and practical implications, limitations of this study, and future research directions.

## Humor and community embeddedness in the context of conservation of resources theory

According to the COR theory, individuals have an overall tendency to strive for, attain, accumulate, and maintain resources that are valuable to them, and that accumulated resources are necessary to achieve important goals (Hobfoll, 2001; Hobfoll et al., 2018). Career satisfaction is one such desirable outcome that requires an enriched pool of resources such as social support, confidence, networking, etc. (Hirschi et al., 2018). However, these resources are usually depleted during career transitions (Mäkelä et al., 2011; McNulty and Moeller, 2018). Expatriates may adopt two coping strategies in such scenarios: they can apply accommodative coping, such as reframing cognition through downward comparisons or altering goals; or they can proactively restore and maintain their resource reservoir by re-establishing lost resources or acquiring substitutes (Hobfoll, 2001). SEH plays a significant role in both these ways. Scholars opine that SEH is an intrapsychic resource that helps individuals adopt an alternative perspective in adversity (Martin et al., 2003), as well as a multifaceted and instrumental resource that facilitates accomplishment while mitigating resource deprivation (Cooper, 2008; Cooper et al., 2018; Nguyen et al., 2022). Evidence shows that community embeddedness represents instrumental resources (Kiazad et al., 2015) that improve the career situation in the host nation (cf. Akkaya et al., 2022).

In the following, we will elaborate on the potential pathways through which humor may promote the career satisfaction of partners in DEECs, either directly or indirectly through community embeddedness, by applying principles of COR theory.

## Multifaceted resources for career satisfaction of dual-earner expatriate couples

### Self-enhancing humor as a multifaceted resource

Humor has always been essential to human existence (Fry, 1994; Gervais and Wilson, 2005). Throughout history, hilarity has aided the survival and functioning of our species through its unique expression and physiological effects (Fry, 1994). Humor elicits not only a range of pleasant feelings such as amusement, joy, and interest (Cheng and Wang, 2015; Wijewardena et al., 2017) but also visible, genuine, and contagious facial expressions such as laughter (Martin, 2007; Herring et al., 2011). Mirthful experiences are also known to enhance cognitive functions, such as the activation of the reward system and the linking of distant concepts (Polimeni and Reiss, 2006; Amir et al., 2015). With regard to physical health, laughter and humor have been shown to promote cardiovascular function, muscle activity, pain relief, the immune system, as well as longevity (Savage et al., 2017; Gonot-Schoupinsky et al., 2020). Various favorable behaviors result from hilarity, such as innovation (Zhang and Su, 2020; Zheng et al., 2022) and organizational citizenship behaviors (Yang and Zhang, 2022). Mirthful behavior is one of the most common experiences in everyday life. Research suggests that, on average, a person encounters a dozen laughter incidents per day at home and jokes every 3–5 min per meeting at work (Martin and Kuiper, 1999; Holmes and Marra, 2002).

Martin et al. (2003) define humor styles according to two facets: (1) whether the humor is benign and tolerant or detrimental and destructive, and (2) whether the humor is used to enhance oneself or to enhance relationships with others. Accordingly, the model recognizes four types of humor and finds that self-enhancing humor has an internal function. It helps people handle difficulties and stress through regulating emotions and changing perspectives (Martin et al., 2003). Additionally, it can support social connections by promoting interpersonal goals like gaining support from others (Martin et al., 2003). Put succinctly, SEH is a multifaceted resource that can mitigate resource loss through mindset change (i.e., accommodative coping). At the same time, it supports resource recovery (i.e., proactive coping) by alleviating negative affectivity and generating instrumental resources to support action orientation.

### Career satisfaction of dual-earner expatriate couples as an outcome of self-enhancing humor

In the literature, career success is generally conceptualized as objective and subjective (Briscoe et al., 2006). Objective career success includes objectively observable indicators (e.g., salary, promotion, and job level) that typically evaluate one’s career against social norms (Spurk et al., 2019). There is a lack of research on the career success of DEECs. The available evidence suggests that one partner, typically the woman, experiences a decrease in job level, referred to as an “occupational penalty” (Ballarino and Panichella, 2018). Career satisfaction (i.e., subjective career success) captures the perceived satisfaction with one’s career achievements, which is a personal experience (Eby et al., 2003; Ng et al., 2005; Briscoe et al., 2021). Career satisfaction appears to assume greater significance than objective career success for partners in DEECs, especially for those who are more disadvantaged (e.g., women, tied movers) (Kierner, 2018; McNulty and Moeller, 2018; Kanstrén and Suutari, 2021). Their frequent disappointments are primarily ascribed to their inability to pursue their desired profession and develop their career capital, rather than to salary or promotion opportunities (Kierner, 2018; McNulty and Moeller, 2018; Kanstrén and Suutari, 2021).

Evidence shows that one partner’s career dissatisfaction often stems from their sacrifices to support the other’s work (Känsälä et al., 2015; Kanstrén and Suutari, 2021). When one partner devotes most of their time and effort to work, their presence and contribution at home decreases, thus impelling the other to compensate and stymieing their career (Mäkelä et al., 2011; Känsälä et al., 2015; Kanstrén and Suutari, 2021). Notably, this situation is more prevalent and detrimental in the context of international relocation, although domestic working couples may face the same problem (Ballarino and Panichella, 2018; Sánchez-Domínguez and Guirola, 2021). As a case in point, the lack of access to childcare systems and support from nearby family members also forces them to sacrifice their career trajectories for caregiving responsibilities (e.g., taking part-time or temporary jobs, declining business travel) (Fischlmayr and Kollinger, 2010; Kanstrén, 2021; Sánchez-Domínguez and Guirola, 2021). Difficulties in career advancement are caused by both external factors (e.g., limited networks, lack of language skills), intra-couple factors (e.g., lack of partner support in career choices and household responsibilities), and personal factors (e.g., external locus of control, negative attitudes and emotions) for those whose caregiving responsibilities are less burdensome (e.g., couples with older children/no children) (Mäkelä et al., 2011; Känsälä et al., 2015; McNulty and Moeller, 2018). Put differently, DEECs’ career situations are typically associated with resource losses that dampen the achievement of desired career goals.

In this light, SEH is likely to help expatriates in DEECs overcome their resource deprivation and thrive in their careers in two ways. First, an internal and perceptual outcome, namely, career satisfaction, is likely to stem from the coping functions of SEH, whereby expatriates can maintain an optimistic view of career situations by transforming challenges into hilarious stimuli (Martin et al., 2003; Mesmer-Magnus et al., 2012; Scheel et al., 2016). This mechanism is a “passive” way to increase feelings of satisfaction in careers (i.e., accommodative coping), given that individuals merely reshape their perceptions of it as opposed to acting to restoring their resource reservoir (Hobfoll, 2001). Second, due to its inherited outcomes that either mitigate resource losses or facilitate resource gains, SEH is generally considered an adaptive coping mechanism (Cooper et al., 2018). Specifically, partners in DEECs with SEH are less likely to experience negative emotions (e.g., anger) and mental health problems (e.g., depression and anxiety) when faced with career difficulties (Martin et al., 2003). This enables them to maintain their resilience in pursuing work aspirations and their determination to accumulate career resources until they achieve their goals (Cheng and Wang, 2015). Then, SEH helps individuals regain the lost career resources such as self-esteem (Martin et al., 2003) and occupational self-efficacy (Scheel et al., 2016), or to generate alternative resources such as social bonds and support in the host country (Martin et al., 2003; Cooper, 2008). For instance, through self-efficacy, they are able to engage in successful entrepreneurship when working in the previous profession is not feasible (McNulty and Moeller, 2018). The benefits of SEH further lie in the pleasant emotions (e.g., joy, happiness, amusement) (Cheng and Wang, 2015; Wijewardena et al., 2017), which are proposed to enhance cognitive functions (e.g., creativity) and personal development (e.g., language) (Chen et al., 2019; Gonot-Schoupinsky et al., 2020). These resources promote perceived career fulfilment by facilitating the adoption of new career pathways (McNulty and Moeller, 2018; Kanstrén and Suutari, 2021). SEH also provides a sense of self-confidence, self-sufficiency, and self-control (Martin et al., 2003), which may lead to higher perceived confidence in taking career steps. Therefore, we expect SEH to be an antecedent of career satisfaction among partners in DEECs.

*Hypothesis 1: The SEH of each partner in DEECs is positively associated with their career satisfaction.*

### Community embeddedness as a mediator between self-enhancing humor and career satisfaction

#### Using humor to create and deepen community embeddedness

In addition to career accomplishment, host community embeddedness is another essential resource that contributes to the partners’ effectiveness in the host country (Horak and Yang, 2016). Community embeddedness represents the off-the-job dimension of job embeddedness, the accumulated forces that keep individuals in their jobs (Mitchell et al., 2001). The immersion of individuals in their community is inclusive of three elements: links, fit, and sacrifice (Mitchell et al., 2001). Links refer to employees’ connections to their community, including social networks, friends, association memberships, and home ownership (Mitchell et al., 2001). Community fit describes their perceived comfort and affinity with the culture, climate, and way of life of the host society (Mitchell et al., 2001). Lastly, their expected loss for leaving the embedded community signifies the sacrifices, which may be intangible (e.g., perceived security, safety, and respect) or tangible (e.g., social security benefits, and pension) (Mitchell et al., 2001). In general, community embeddedness denotes a pool of resources acquired and maintained by individuals within the locality (Kiazad et al., 2015), which can be reinvested to enhance the work functions of expatriates [e.g., performance and Organizational citizenship behaviors (OCBs)] (Andresen, 2015).

Regarding career situations, resources available in the home country community (e.g., social support) are often lost after an international relocation (Agha-Alikhani, 2016). This requires the reservoir’s reestablishment in the host country. While individuals can change their viewpoint to meet career desires (i.e., accommodative coping), immersion in a new host society and community requires proactive coping and investment of instrumental resources (i.e., proactive coping). SEH acts as a resource in myriad ways to foster expatriates’ interpersonal relationships, and group cohesion in the community (Cooper, 2008). It is clear that individuals with SEH can often relieve themselves of stressors and view their adversities (e.g., culture shock, discrimination, and hostility) from a comic perspective (Martin et al., 2003). As this implants pleasurable affectivity in their social interactions and nurtures their friendships (Jones et al., 2021), they are pleasant to be around (Mesmer-Magnus et al., 2012). Similarly, their SEH attracts like-minded individuals in the host community, thus cultivating a social network (links), and increasing fit with locals (Cooper, 2008). As SEH is often associated with benevolence such as empathy (Hampes, 2010), and social support (Martin et al., 2003; Zhao et al., 2014), partners in DEECs are likely to perceive their immediate social groups as supportive, thereby increasing fit in the local society. Lastly, expatriates may confront a higher risk of discrimination and hostility, thus impeding their immersion in the host community (Nguyen, 2022). SEH is a tool used to eliminate the social barriers between themselves and the locals (Cooper, 2008), for instance, by turning hostile or conflictual incidents into comic stimuli. In this manner, SEH can enrich their links with the locals and their perceived safety and respect in the host country (i.e., sacrifice). In summary, SEH acts as an instrumental resource to deepen expatriates in DEECs’ community embeddedness.

#### Community embeddedness as a resource for building career satisfaction

The various threats of resource loss associated with the social conditions of expatriate couples include non-recognition of qualifications, language deficiency, discrimination, precarious working conditions, and a lack of network and social support (Mäkelä et al., 2011; Kil et al., 2018), which hinder the achievement of career goals. Because becoming embedded compensates for the lack of these social resources, it is a proactive way to build career satisfaction in the local community. To illustrate, although a relocation often deprives expatriates of the support of the wider family, friends in the host community can help with administrative tasks (e.g., taxes and insurance), language, emotional strains, and child rearing (Agha-Alikhani, 2016). These resources allow expatriates in DEECs to devote their energy, effort, and mentality to developing their careers. In addition, social relationships and acculturation enable partners to develop their career capital, including their professional network, soft skills, and international competencies (Kanstrén and Suutari, 2021), which are essential for career satisfaction (Hirschi et al., 2018). Thus, community embeddedness is likely to augment the career satisfaction of partners in DEECs.

In wake of the aforementioned arguments, SEH is likely to influence career satisfaction both directly and indirectly through deepening community embeddedness. For instance, the network and social support resulting from their humorous characteristics help in identifying suitable employment/business opportunities and developing career capital (Beaverstock, 2002; Kanstrén and Suutari, 2021). On the basis of the arguments outlined above, we predict that:

*Hypothesis 2: The positive relationship between the SEH of each partner in DEECs and their career satisfaction is mediated by each partner’s community embeddedness.*

### Crossover effects of self-enhancing humor on the partner’s community embeddedness and career satisfaction

Crossover effects explicate the process whereby characteristics, resources, or strains are transferred between individuals in shared environments or close relationships (Demerouti, 2012). In COR theory, resources exist in caravans, and not individually. Thus, crossover denotes the transfer of resources from one person to another within resource caravans. This crossover effect occurs under appropriate conditions (i.e., passageways) (Hobfoll et al., 2018).

The wheel of humor model illustrates passageways through which a partner’s SEH influences not only their own community embeddedness but also that of their partner, as well as career satisfaction. This model posits that individuals experiencing humorous events can initiate and foster a humor-supportive environment that stimulates pleasant emotions at the group level, subsequent mirthful episodes, and a long-term humorous climate (Robert and Wilbanks, 2012). This hilarious and playful climate also influences the behavior of individuals within it, even if they do not initiate the humor (Robert and Wilbanks, 2012). Generally, the literature supports the model with concrete empirical evidence. As a case in point, research demonstrates that humor occurring within team interactions triggers subsequent mirthful events, creates humor patterns at the team level, and eventually promotes team performance (Lehmann-Willenbrock and Allen, 2014). Similarly, leader humor increases subordinates’ innovative behavior by increasing their work engagement (Zhang and Su, 2020) or through psychological empowerment (Zheng et al., 2022). Leaders’ use of humor can have a positive impact not just in the workplace, but also in subordinates’ personal lives. Their humorous behavior can enhance job satisfaction and balance between work and family, leading to increased satisfaction in their partners’ marriages (Horn et al., 2019). Studies have shown that a person’s sense of humor can have a positive impact on their partner’s emotions within a relationship. An individual’s sense of humor can create a cheerful atmosphere in the home (Horn et al., 2019).

In this sense, we expect SEH-triggered resources to cross over from one partner to another among DEECs. A mirthful expatriate’s partner is likely to benefit from a hilarious home environment (i.e., resource caravan passageways), where they can employ resources such as positive affectivity (Horn et al., 2019), optimism (Scheel et al., 2016), self-efficacy (Scheel et al., 2016), and persistence (Cheng and Wang, 2015). Therefore, the SEH of one partner is likely to have the same impact on the other partner’s career satisfaction as it does on the humorous partner himself or herself (cf. Hypothesis 1).

*Hypothesis 3: SEH of each partner in DEECs is positively associated with the other’s career satisfaction.*

Similarly, a humor-supportive environment tends to promote hilarious experiences, well-being, and pleasant emotions of partners in DEECs (Robert and Wilbanks, 2012; Horn et al., 2019), paving the way for their social bonding and relationships (Cooper, 2008). Feelings of amusement are known to further increase persistence (Cheng and Wang, 2015) and cognitive functions (Gonot-Schoupinsky et al., 2020; Nguyen et al., 2022), which play a crucial role in the accomplishment of goals such as learning a new language and acquiring information about the culture and way of life in the host country. Thus, we expect one partner’s SEH to facilitate the other partner’s embedding into a host community in the same way that it increases that of the mirthful partner, and, subsequently influences the other partner’s career satisfaction indirectly (cf. Hypothesis 2).

*Hypothesis 4: The positive relationship between the SEH of each partner in DEECs and the other partner’s career satisfaction is mediated by the other partner’s community embeddedness.*

### Gender as a moderator of the crossover effects of self-enhancing humor on the partner’s community embeddedness and career satisfaction

The wheel of humor model typically involves the contagion of humor-induced affect (e.g., amusement) and expressions (e.g., laughter) between individuals (Robert and Wilbanks, 2012). While humor tends to elicit intense, distinctive, and lucid emotions and displays (e.g., laughter) (Herring et al., 2011), it is possible for the propensity to “catch” and harmonize with these responses differ by gender. According to the literature, women are more likely than men to pay attention to others’ emotions, to interpret facial and vocal expressions more accurately, and to engage in more mimicry of emotional expressions, particularly when it comes to long-term interactions (Doherty et al., 1995; Sonnby-Borgström et al., 2008; Magen and Konasewich, 2011). Sonnby-Borgström et al. (2008) propound that gender differences in contagious susceptibility stem from the predilection to regulate emotions. Men are more likely than women to suppress or mask their emotional expressiveness due to gender stereotypes of masculinity (Sonnby-Borgström et al., 2008). Additionally, women’s sensitivity to the emotions of others may be related to their primary survival strategy of relying on social support and cooperation in the face of threat (i.e., “tend-and-be-friend”) (Sonnby-Borgström et al., 2008). This distinguishes them from men’s tendency to “fight-or-flight” (Sonnby-Borgström et al., 2008). Therefore, female partners in DEECs are more likely than men to notice their partner’s humor experiences and reactions, thus accelerating the shared humor patterns and environment. We expect women’s contagious tendency to strengthen the effects of their partner’s humor on their community embeddedness and career satisfaction as compared to those of men.

*Hypothesis 5: The positive crossover relationships between one partner’s SEH and the other partner’s (a) community embeddedness and (b) career satisfaction are moderated by gender, with the relationship being stronger for female than for male partners.*

Hypothesis 5 explores the impact of gender on the relationship between SEH and career satisfaction (as outlined in Hypothesis 3) as well as on the initial stage of the mediating process that links the two (as described in Hypothesis 4). Our hypothesis holds that the mediation outlined in Hypothesis 4 and the moderation described in Hypothesis 5 will occur simultaneously. Consequently, we propose a moderated mediation hypothesis. Based on the possibility that SEH’s association with the partner’s community embeddedness might vary based on gender (Hypothesis 5), we anticipate a similar result for its relationship with career satisfaction, that is:

*Hypothesis 6: Gender moderates the positive indirect crossover relationship of one partner’s SEH with the other’s career satisfaction through community embeddedness, with the relationships being stronger for female than for male partners.*

## Methodology

### Data collection and sample description

Our longitudinal dyadic data include 109 cohabiting and heterosexual DEECs in Europe. Eligibility criteria include: (1) both partners reside in a country other than their country of upbringing, (2) the partners live together, and (3) both partners are employed in their country of residence. Participants were recruited through Facebook groups, a consumer panel, and Prolific (i.e., an online data collection platform). Since the call was published to the different audiences on these platforms, it is not possible to obtain any information on how many eligible participants accessed the online survey. The couples in the study completed the questionnaire one after the other. We asked them to keep their answers confidential from the others until the survey’s completion. After the couples concluded their questionnaire, they received our thanks and a monetary reward (8.40 ₤), except for those on Facebook who volunteered to be our participants without monetary gifts. The first wave of data collection occurred between July 2020 and June 2021. In this wave, 770 couples participated in the survey, of which 220 were eligible and met the quality standards (e.g., response time, and consistency between responses) [see Meade and Craig (2012)]. To answer the second questionnaire, the second wave of data collection took place by sending invitations to the 220 couples after 1 year. Notably, 109 couples returned completed questionnaires, giving an overall response rate of 49.5%. In terms of data sources, the response rate was 100% for Facebook, 49.8% for Prolific, and 28.6% for the consumer panel. These differences could be ascribed to external issues rather than participants’ interest in our research, as many eligible participants from the first wave were not available on Prolific and the consumer panel when we conducted the second wave. Nevertheless, our analysis does not reveal any differences between the data sources for the variables included in this study.

The study’s participants resided in 14 European countries, mainly in the United Kingdom (45%), Spain (14%), and Germany (12%). They came from various countries around the world (49 for men, and 51 for women). Within the sample, 80.7% of the men and 70.6% of the women were employed full-time, 11% of the men and 22.9% of the women were employed part-time, and the rest (8.3% of the men, 6.4% of the women) were self-employed in the host nations. In addition, 86.7% of the participants were highly educated (37.6% bachelor, 40.4% master, and 8.7% doctorate). The remainder had completed post-secondary non-tertiary or short-cycle tertiary (11%), upper secondary (1.4%), lower secondary (0.5%), and primary education (0.4%). A total of 90.4% of the participants were self-initiated expatriates, 7.8% were assigned expatriates, and the rest (1.8%) did not specify their expatriation mode. Most participants met their partners before relocating to the host country (68.8%). Around half of these stated that the woman was the main driver in their decision to relocate (46.7% of men and 51.4% of women), around a sixth stated that the man played this role (13.3% of men and 17.6% of women) whereas about a third pointed out that they both influenced the decision equally (40% of men and 31.1% of women).

### Measures

Participants rated each item on the following scales using a Likert scale ranging from 1 (strong disagreement) to 5 (strong agreement) unless otherwise specified. The levels of all variables were analyzed for the data.

#### Main variables

*Self-enhancing humor* was measured by the 8-item validated scale developed by Martin et al. (2003) and collected in the second wave. Sample items include “Even when I’m by myself, I’m often amused by the absurdities of life” and “If I am feeling sad or upset, I usually lose my sense of humor” (reversed score). The scale obtained satisfactory reliability (Cronbach’s α = 0.89 for men and 0.88 for women).

*Host country community embeddedness* was measured by the scale developed and validated by Mitchell et al. (2001), as adapted to the expatriation context by Tharenou and Caulfield (2010), and collected in the first wave. The scale comprised eight items, of which the items related to the fit and sacrifice dimensions were rated on a Likert-scale. An example is “I really love the place where I live.” In addition, participants were asked to provide information about their community links, such as “How many children are living with you now?” and “How many of your close friends live nearby?” As recommended by Mitchell et al. (2001), we standardized the latter item for further analysis. The scale yielded good internal consistency (Cronbach’s α = 0.81 for both men and women).

*Career satisfaction* was conducted during the second wave and assessed using a 5-item career satisfaction scale established by Greenhaus et al. (1990). Sample items are “I am satisfied with the progress I have made toward meeting my goals for income” and “I am satisfied with the progress I have made toward meeting my goals for the development of new skills.” The scale obtained satisfactory reliability (Cronbach’s α = 0.94 for both men and women).

#### Control variables

The literature suggests that education and personal initiative influence individual career satisfaction (Ng et al., 2005), whereas language proficiency, length of stay in the host country, and personal initiative affect international workers’ community embeddedness (Ren et al., 2014; Yunlu et al., 2018; Nguyen and Andresen, 2021). Thus, we controlled for these variables in our analysis. Host country language proficiency was rated from 1 (“poor”) to 4 (“excellent”). To measure personal initiative, we used the 7-item scale developed by Frese et al. (1997). Example item: “I actively attack problems.” (Cronbach’s α = 0.87 for men and 0.84 for women). Data for these variables were collected at wave 1.

### Data analysis

We used the Actor-Partner Interdependence Model (APIM) (Kenny et al., 2006) and Mplus version 8.7 to analyze our data. In addition to controlling for the non-independence between partners, the APIM allows us to simultaneously examine the influences of each partner’s factors on their own outcomes (*X*_1_ → *Y_1_; X_2_* → *Y*_2_) (actor effects) and on the partner’s outcomes (*X*_1_ → *Y_2_; X_2_* → *Y*_1_) (partner or crossover effects) (Kenny et al., 2006). In our study, *H1* and *H2* describe the actor effects of SEH on career satisfaction and the mediation by community embeddedness. At the same time, *H3* and *H4* indicate the partner effects with the same predictors and outcomes. The extended version of APIM–the APIMeM–makes it possible to test mediation effects in the dyadic model using Structural Equation Modeling (SEM) techniques (Ledermann et al., 2011). The main components of APIMeM include three pairs of measured variables [i.e., predictors (*X*_1_; *X*_2_), mediators (*M*_1_; *M*_2_), outcomes (*Y*_1_; *Y*_2_)], and two pairs of residual terms of mediators (*E*_1_; *E*_2_), and outcomes (*E*_3_; *E*_4_) (Ledermann et al., 2011). *M*_1_ and *M*_2_ mediate relationships between predictors and outcomes in a dyadic model in several ways. In our model, the mediators (community embeddedness) are proposed to mediate both actor effects (*X*_1_ → *M*_1_ → *Y*_1_; *X*_2_ → *M*_2_ → *Y*_2_), and partner effects (*X*_1_ → *M*_2_ → *Y*_2_; *X*_2_ → *M*_1_ → *Y*_1_). The correlations within each couple indicate the non-independence between the variables in that couple. If the correlation is significant (e.g., between *X*_1_ and *X*_2_), APIM ensures that the regression equation is done involving either variable (e.g., *X*_1_ → *M_1_*) while controlling for the other variable (e.g., *X*_2_) (Kenny et al., 2006). It is important to ascertain whether the dyads are distinguishable or indistinguishable in order to analyze using APIM (Kenny et al., 2006). We applied the model for distinguishable members according to their sex since we want to test the difference between men and women in their partner effects (*X*_1_ → *Y_2_; X_2_* → *Y_1_; X_1_* → *M*_2_ → *Y*_2_; *X*_2_ → *M*_1_ → *Y*_1_). Lastly, we examined the moderation of gender on partner effects by applying the Wald test, which detects the equality of multiple logit models (Liao, 2004).

## Results

**FIGURE 1:**
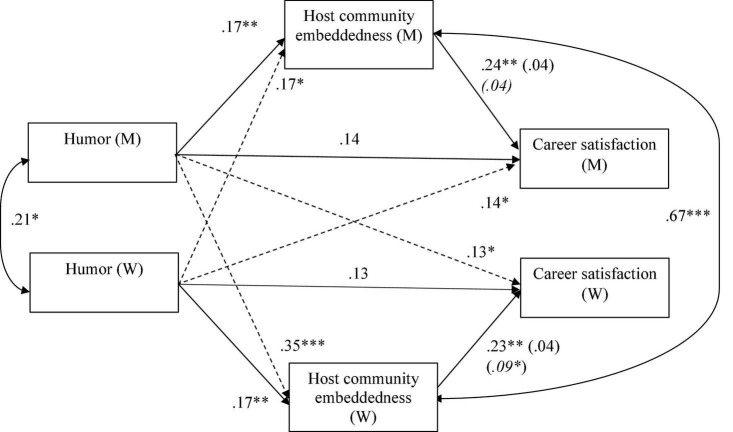
Research results. ________: Actor effects. - - - - - -_: Partner effects. *p < 0.05; ^**^p < 0.01; ^***^p < 0.001. Estimates of partner’s mediation effects are marked in *italics* in brackets. The actor paths was controlled for education, personal initiative, language proficiency, and duration in the host country.

Table 1 illustrates the descriptive statistics of the variables in our study. Table 2 demonstrates the comparisons of our alternative models. In the base model, all paths were freely estimated. The model fit did not decrease when we constrained the actor paths across gender, suggesting that actor effects did not differ by partner gender. However, constraining partner paths significantly worsened the model fit: Changes in the χ^2^ value and degrees of freedom produced a significant result (*p* < 0.05). Thus, the partner effects were likely to vary between men and women. Subsequently, we examined the model where the partner effects of SEH on community embeddedness were freely estimated. This significantly improved the model fit (Δχ^2^/Δ*df* = 0.03, *p* < 0.05), suggesting that only the partner effects of SEH on community embeddedness varied by gender, leading to our final model. As recommended by Kenny et al. (2006), we examined the model fit using the Chi-Square Test, Root Mean Square Error of Approximation (RMSEA) (Steiger and Lind, 1980), and Comparative Fit Index (CFI) (Hu and Bentler, 1998). The goodness of fit criteria included a non-significant Chi-Square Test, RMSEA less than or equal to 0.6, and CFI of at least 0.95 (Hu and Bentler, 1998; Kenny et al., 2006). Our model demonstrated an excellent fit to the data: *χ^2^* (52) = 51.4, *p* = 0.50; RMSEA = 0.00; CFI = 1.00. Figure 1 presents a summary of our research model’s results. Overall, our model explained 25.8% of the variance in men’s career satisfaction, 22.1% in women’s career satisfaction, 21.6% in men’s community embeddedness, and 25.3% in women’s community embeddedness.

**TABLE 1:** Means, standard deviations, and correlations.

Variables	Mean	SD	(1)	(2)	(3)	(4)	(5)	(6)	(7)	(8)	(9)	(10)	(11)	(12)	(13)
1. Education (M) (T1)	6.42	1.04													
2. Education (W) (T1)	6.39	0.82	0.32**												
3. Personal initiative (M) (T1)	3.92	0.72	0.07	-0.21*											
4. Personal initiative (W) (T1)	3.85	0.68	0.08	0.19	0.19										
5. Language (M) (T2)	3.28	0.95	-0.09	0.09	-0.06	-0.09									
6. Language (W) (T2)	3.33	0.85	-0.13	0.10	-0.13	-0.00	0.52**								
7. Duration in the host country (years) (M) (T2)	7.89	7.54	-0.05	-0.01	0.05	0.09	0.29**	0.15							
8. Duration in the host country (years) (W) (T2)	6.88	6.39	0.02	-0.03	0.01	0.03	0.14	0.20*	0.77**						
9. Self-enhancing humor (M) (T2)	3.15	0.87	-0.21*	-0.15	0.19	0.08	0.06	-0.05	-0.01	-0.10					
10. Self-enhancing humor (W) (T2)	2.99	0.89	-0.05	-0.19	0.19	0.24*	-0.01	0.07	0.01	0.07	0.21*				
11. Host community embeddedness (M) (T1)	2.43	0.59	0.02	-0.15	0.32**	0.16	0.03	-0.06	0.28**	0.26**	0.26**	0.22*			
12. Host community embeddedness (W) (T1)	2.44	0.60	-0.02	-0.17	0.23*	0.16	-0.07	-0.01	0.14	0.25**	0.38**	0.24*	0.71**		
13. Career satisfaction (M) (T2)	3.66	0.98	0.09	-0.20*	0.38**	-0.04	0.07	-0.03	0.21*	0.07	0.30**	0.29**	0.33**	0.26**	
14. Career satisfaction (W) (T2)	3.40	1.02	0.07	0.11	0.03	0.29**	-0.06	-0.03	0.01	0.00	0.20*	0.19	0.18	0.36**	0.18

*p < 0.05; **p < 0.01.

**TABLE 2:** Model comparison.

Model	χ^2^	Degrees of freedom	*p*-value	RMSEA	CFI	AIC	Δ χ^2^ *p*-value
1. All paths freely estimated	48.752	48	0.44	0.012	0.996	2,481.80	
2. Actor paths constrained across gender	50.968	51	0.47	0.000	1.000	2,478.02	0.53
3. All partner paths constrained	56.159	53	0.36	0.023	0.981	2,479.21	0.02
4. Final model (partner path: SEH → community embeddedness was freely estimated)	51.424	52	0.50	0.000	1.000	2,476.48	0.03

### Actor effects

The relationship between SEH and career satisfaction was insignificant for both men (β = 0.14, 95% CI [−0.01, 0.28], *p* = 0.07), and women (β = 0.13, 95% CI [−0.01, 0.27], *p* = 0.06). The results, therefore, rejected *H1.* In contrast, community embeddedness significantly predicted career satisfaction for both women (β = 0.23, 95% CI [0.07, 0.40], *p* < 0.01) and men (β = 0.24, 95% CI [0.07, 0.40], *p* < 0.01). According to the findings, SEH was positively associated with community embeddedness for both men (β = 0.17, 95% CI [0.06, 0.28], *p* < 0.01) as well as women (β = 0.17, 95% CI [0.06, 0.27], *p* < 0.01). We used 10,000 bootstrapping estimates to test the mediating effect of community embeddedness on the relationship between SEH and career satisfaction. The 95% confidence intervals did not contain 0 (β = 0.04, 95% CI [0.01, 0.10], *p* = 0.05), supporting *H2*.

### Partner effects

Women’s SEH was found to be significantly associated with men’s career satisfaction (β = 0.14, 95% CI [0.00, 0.27], *p* < 0.05). Similarly, the relationship between men’s SEH and women’s career satisfaction was significant (β = 0.13, 95% CI [0.00, 0.25], *p* < 0.05). Therefore, *H3* was supported. Men’s SEH turned out to be a strong predictor of women’s community embeddedness (β = 0.35, 95% CI [0.22, 0.46], *p* < 0.001), and women’s SEH significantly predicted men’s immersion in the community (β = 0.17, 95% CI [0.02, 0.31], *p* < 0.05). The results from the 10,000 bootstrapping confidence interval tests showed that men’s SEH had a significant impact on women’s career satisfaction through women’s level of community involvement (β = 0.09, 95% CI [0.02, 0.18], *p* < 0.05). However, men’s community embeddedness did not mediate the relationship between women’s SEH and men’s career satisfaction (β = 0.04, 95% CI [0.00, 0.11], *p* = 0.12). The results partially supported *H4*.

We employed the Wald test to examine the moderation effect of gender on partner paths. Results showed that gender had a significant moderation effect on the linkage between SEH and community embeddedness [β = 0.17 vs. β = 0.35, Wald χ^2^ (*df* = 1) = 4.779, *p* < 0.05], supporting *H5a*. However, gender did not moderate the direct path from SEH to career satisfaction [β = 0.14 vs. β = 0.13, Wald χ^2^ (*df* = 1) = 0.457, *p* = 0.49], thereby rejecting *H5b*. The one-tailed Wald test reveals that the mediation effects of community embeddedness on the relationship between SEH and career satisfaction were stronger for women than men [β = 0.04 vs. β = 0.09, Wald χ^2^ (*df* = 1) = 3.384, *p* < 0.05], thereby supporting *H6*. Figure 1 illustrates the results of the research model.

## Discussion

In congruence with the results of this study, social and multifaceted resources played an important role in the career satisfaction of partners in DEECs. The partners generally utilized their own SEH to enhance their community embeddedness, resulting in increased career satisfaction. However, their use of humor did not significantly enhance their career success perception. Conversely, a partner’s humor had a direct positive impact on the other partner’s community embeddedness and career satisfaction. Additionally, there were gender differences in how partners utilized their partner’s humor to deepen their integration in the community: Women benefited more than men from the indirect effects of their partner’s humor on career satisfaction through community embeddedness. The following section discusses further implications of the results of this study.

### Theoretical and research implications

The resource caravan passageway principle posits that the crossover effects occur through empathy, the transmission of experiences, and shared stressors (Hobfoll, 2001). Generally, these are rational pathways that involve individual awareness and relative control over the process. By complementing COR theory with the wheel model of humor, this study illustrates that resource transmission can occur without people’s intention or awareness and that cycles of transmission can reinforce the passageways of resource caravans (Robert and Wilbanks, 2012). This opens up opportunities for subsequent investigations to delve into the crossover-influence of humor and emotional-related aspects in collective and duo contexts.

This study demonstrates that taking a positive approach has notably increased our knowledge of career satisfaction among DEECs. While current research has addressed essential factors that undermine couples’ career satisfaction, it is necessary and beneficial to identify potential resources for this outcome. Community embeddedness and humor have been among the neglected resources in studies on the careers of expatriate couples. Using COR theory (Hobfoll, 2001), we showed that humor and community embeddedness are two resources for DEECs to achieve their career goals. These resources exist outside the work context, suggesting that resources in private life may contribute significantly to couples’ career satisfaction. While previous research has often portrayed DEECs’ career satisfaction as suffering (cf. Kupka and Cathro, 2007), this study shows that playful, entertaining, and endearing feelings at home are valuable contributors to resolving their career struggles. This calls for further research into the various non-work resources that facilitate dual-career satisfaction among expatriate couples.

The marginal findings regarding the relationship between expatriates’ SEH and career satisfaction suggest that altering perceptions of career circumstances with SEH may have some positive impact. However, the key to enhancing career satisfaction is by fostering connections with the local community and acquiring tangible resources (cf. McNulty and Moeller, 2018). This further explains why gender did not differentiate the direct crossover effect of humor on career satisfaction. We suggest that the direct effect of humor on career satisfaction should not be predicated on gender differences, as it primarily occurs through perceptual change, an intrapersonal process (cf. Ng et al., 2005). Nevertheless, further studies are needed to test the interpretation of our results. Another reason may be the relatively small sample size of our study, which may hinder the detection of existing effects. Further studies can replicate our model with a larger sample size to examine the validity of these findings.

The study also found that SEH-generated resources transcend within DEECs and boost career satisfaction for both partners, regardless of who has this attribute. This highlights the significance of having a positive characteristic like humor and how a partner’s SEH can affect career satisfaction in expatriate couples. Our research suggests that the resources and functions produced by a humorous partner may be sufficient to provide benefits to both (e.g., health benefits, cognitive functions, persistence, and social bonds), which in turn increase the sense of career accomplishment and community immersion.

Finally, men and women used resources from their partner’s SEH for their community embeddedness in different ways, which in turn diverged the influence of their partner’s humor on their career satisfaction. Specifically, women’s partners’ mirth was more likely to foster women’s community embeddedness than men’s. This was probably attributed to women’s heightened tendency to capture their partner’s mirthful expressions and feelings and to converge with pleasant and lively emotions. Furthermore, research suggests that community embeddedness is more demanding for female than male partners, for instance in terms of local social networks (Martinovic et al., 2015). This may be due to women’s more salient domestic roles (e.g., child-rearing) than men’s (Känsälä et al., 2015), as well as their challenges in building community embeddedness, may increase the importance of the humor climate initiated by their SEH partners. Further research is needed to explore gender differences in the use of partner resources among expatriate couples, and the consequences of these differences.

The findings provide valuable insights for research on the dual career satisfaction of expatriate couples. First, they suggest that their career achievement is also a *dyadic* outcome in which each partner influences the other’s career satisfaction in different ways. We can enrich our knowledge and develop appropriate interventions for their career management by identifying both the ways in which crossover occurs and its consequences. This leads to the second implication, which is that dyadic methods of analysis, such as APIM, may be necessary for research on the careers of couples within the expatriate population. Currently, the utility of dyadic analysis is rarely, if ever, used in research on the work of expatriate couples.

### Practical implications

This study outlines several ways in which organizations can support DEECs’ career satisfaction. First, employers can help DEECs become embedded in the host community. To illustrate, they can organize language training, informal social networking events, cultural tours, and/or provide information about the host country’s way of life, legal and political systems, and/or social support (e.g., psychological coaching, counseling, support groups, etc.). They can liaise with the youth and family services to provide counseling on family settlement, relationships, administrative tasks, parental benefits, and child-rearing.

Second, to support the career achievements of expatriates in committed relationships, organizations can take advantage of self-enhancing humorous partners. This can be a consideration when selecting expatriates for international assignments. Organizations can also actively implement humor training for expatriate couples, by organizing playful and hilarious events for expatriate families. For instance, the “seven humor habits program” has been shown to be effective in increasing hilarity and its long-term benefits in individuals (McGhee, 2010; Crawford and Caltabiano, 2011). This intervention can be a part of career management for expatriate couples to facilitate their career progression.

### Limitations and directions for future research

No study is impervious to limitations, and our study is no exception. First, the independent variable SEH was measured in the second wave. The justification is that self-enhancing comic style is a stable trait that generally does not change over time (Mesmer-Magnus et al., 2012; Hunter et al., 2016), with a few exceptions, such as those who receive systematic intervention in clinical settings (Kugler et al., 2021). SEH reinforced the results of our analysis despite the unusual order of measurement. Nevertheless, future research should measure SEH in multiple waves to verify causal effects. Second, the sample contains a majority of DEECs consisting of self-initiated expatriates and only a small proportion consisting of assigned expatriates. This could lead to a bias in our results, as self-initiated expatriates experience are likely to rely more on personal resources because they have less organizational support. Including only DEECs residing in Europe may also limit the generalizability of our findings to a broader range of populations. Scholars can replicate our study in other contexts and with different samples to examine whether contextual factors (e.g., childcare system, gender norms) and individual factors (e.g., expatriate mode) moderate our research model. Third, due to the predetermined testing of gender differences, we did not include homosexual couples in the sample of this study. Given their increasingly prominent role in the international talent pool (McPhail et al., 2016), it would be insightful to look at their careers from a dyadic career lens and explore the within-couple factors that influence their dual satisfaction. Finally, the influence of children on the career satisfaction of expatriate partners was beyond the scope of this study. Nonetheless, expatriate parents’ career outcomes are likely to cross over to their children’s conditions and vice versa [see van der Zee et al. (2007)]. Thus, the influences of children’s humor on expatriate parents’ careers may be another promising avenue of research.

## Conclusion

Our research indicates that humor has a positive impact on the career satisfaction of partners in DEECs through connectedness with the local community. Additionally, one partner’s SEH positively affects the other’s career satisfaction. Men’s careers were largely impacted by their own SEH, with some additional benefits from their partner’s humor. For women, their involvement in the community was primarily influenced by their partner’s SEH, leading to improved career satisfaction.

## Data availability statement

The datasets presented in this article are not readily available because the participants of this study did not give written consent for their data to be shared publicly. Requests to access the datasets should be directed to the authors.

## Ethics statement

The studies involving human participants were reviewed and approved by the Ethikrat, Universität Bamberg. The patients/participants provided their written informed consent to participate in this study.

## Author contributions

AN contributed to the general concept, data collection, formulation of hypothesis, analysis, and writing of the manuscript. MA contributed to funding, data collection, general concept, formulation of hypothesis, and writing of the manuscript. All authors contributed to the article and approved the submitted version.
